# Coupled transcriptome and proteome analysis of L3 and L4 developmental stages of *Anisakis simplex* s. s.: insights into target genes under glucose influence

**DOI:** 10.1186/s12864-025-12068-w

**Published:** 2025-09-29

**Authors:** Iwona Polak, Robert Stryiński, Mateusz Maździarz, Lukasz Paukszto, Mónica Carrera, Iwona Bogacka, Elżbieta Łopieńska-Biernat

**Affiliations:** 1 Department of Biochemistry, Faculty of Biology and Biotechnology, University of Warmia and Mazury in Olsztyn, Michała Oczapowskiego 2, 10-719 Olsztyn, Poland; 2 Department of Botany and Evolutionary Ecology, Faculty of Biology and Biotechnology, University of Warmia and Mazury in Olsztyn, Plac Łódzki 1, 10-721 Olsztyn, Poland; 3 Department of Food Technology, Institute of Marine Research, Spanish National Research Council, Eduardo Cabello 6, 36-2018 Vigo, Spain; 4 Department of Animal Anatomy and Physiology, Faculty of Biology and Biotechnology, University of Warmia and Mazury in Olsztyn, Michała Oczapowskiego 2, 10-719 Olsztyn, Poland

**Keywords:** *Anisakis simplex*, Glucose uptake, Transcriptomics, Proteomics

## Abstract

*Anisakis simplex* is a cosmopolitan parasitic nematode of marine organisms with a complex life cycle. Consuming fish infected with its larvae poses a health risk, as the parasites can penetrate gastrointestinal mucosa, damage stomach and intestinal walls, and trigger allergic reactions. The resulting disease is known as anisakiasis. The European Food Safety Authority (EFSA) classifies *A. simplex* as a biohazard. Most of its developmental stages (L3, L4, and adults) occur under anaerobic conditions, and larvae derive energy mainly from saccharides. However, the effect of glucose on L3 and L4 larvae—stages pathogenic to humans—has not been described. This study aimed to identify genes and pathways involved in glucose (10 mg/mL) response through transcriptomic and proteomic analyses of L3 and L4 larvae. Differentially expressed genes (DEGs), long non-coding RNAs (DELs), and differentially regulated proteins (DRPs) were identified. DEGs were involved in cuticle structure, lyase activity, and metabolic processes. Comparing L3 CTR and L4 CTR (control) to glucose-treated samples revealed 1,969 DEGs; 259 overlapped between L4 GLU vs. L3 GLU, (glucose-treated) with 11 showing reversed expression. Additionally, 84 DELs were identified in L3 GLU vs. control, 40 in L4 GLU vs. control, and 163 between glucose-treated L4 and L3. Larval and glucose-specific alternative splicing events were also analyzed. Proteomic analysis revealed 35 DRPs—5 more abundant in L4, 30 in L3. The data reveal that developmental stage exerts a more substantial influence than glucose exposure on gene and protein expression profiles. However, glucose still modulates several pathways related to translation, cytoskeletal remodeling, extracellular matrix (ECM) reorganization, and energy metabolism.

**Supplementary Information:**

The online version contains supplementary material available at 10.1186/s12864-025-12068-w.

## Background

*Anisakis simplex* is a cosmopolitan parasitic nematode of marine organisms characterized by a complex developmental cycle. Eating fish containing larvae can pose a serious health risk, as these parasites can penetrate the mucous membranes of the gastrointestinal tract and cause damage to the stomach and intestinal walls as well as trigger allergic reactions. The disease caused by nematodes of the genus *Anisakis* is known as anisakiasis [1, 2]. According to the European Food Safety Authority (EFSA), *A. simplex* has been classified as a biohazardous organism [3, 4]. Most developmental stages (L3, L4, and adults) of the parasitic nematodes take place under anaerobic conditions and the larvae obtain the majority of their energy from saccharides [5, 6]. The uptake of glucose from the intestinal lumen has not been described in L3 and L4 larvae of *A. simplex*. The organization and structure of digestive system varies between free-living and parasitic species and between developmental stages. These differences result from changes in diet and feeding behavior. In L3 larvae of *A. simplex*, the intestinal lumen is shrunken. Only after the third moult, in L4 larvae, does the intestine become clear and begin to function [7]. It is worth noting is that only these stages have been identified as pathogenic to humans [8]. Glucose uptake and subsequent metabolism play a crucial role in the survival and development of parasitic nematodes. Glucose serves as a primary energy source that drives vital processes and enables adaptation to changing environmental conditions. Previous studies on other parasitic nematodes have shown that metabolism changes markedly in response to glucose availability, including alterations in energy production pathways, stress response mechanisms and biosynthetic activities [9, 10]. However, the specific metabolic pathways affected by glucose uptake in *A. simplex* (s. s.) are not known. To address this gap, this study utilizes a coupled transcriptome and proteome analysis to investigate the molecular basis of the effects of glucose on metabolism at developmental stages L3 and L4 of *A. simplex* (s. s.). This dual approach enables the identification of matching and mismatching patterns between mRNA and protein levels and provides insights into post-transcriptional regulation and metabolic pathway dynamics. Our research addresses several key questions: How do transcriptomic and proteomic profiles differ under the glucose treatment between the L3 and L4 stages? Which metabolic pathways are primarily regulated by glucose uptake? Are there stage-specific adaptations in energy metabolism? Answering these questions will enhance our understanding of the metabolic strategies of *A. simplex* (s. s.) and provide deeper insights into the developmental biology of parasitic nematodes. Notably, the predominant pathway of glucose transport in parasitic nematodes remains unclear, as do the changes in its contribution during larval development.

## Methods

### In vitro culture of A. simplex and treatment with glucose

The study was carried out on the larval stages L3 and L4 of *Anisakis simplex* s. s. (Rudolphi, 1809) Dujardin, 1845. The nematodes in the L3 stage were isolated from Baltic herring (*Clupea harengus* Linnaeus, 1758). All larvae, i.e. 200 individuals, were assessed as belonging to *Anisakis* spp. under the stereomicroscope [11]. Twenty larvae were taxonomically identified by real-time PCR to amplify the ITS region using the Anis Sensitive Sniper RT-PCR kit (A&A Biotechnology, Gdynia, Poland), as previously described [12]. The kit is manufactured upon customer request and is designed to identify the representatives of nematodes from the family Anisakidae found in the Baltic and North Atlantic regions [*A. simplex* (s. s.), *Anisakis pegreffii*, *Pseudoterranova decipiens* (s. s.), *Pseudoterranova krabbei*, *Contracaecum osculatum* (s. s.), and *Hysterothylacium aduncum* (from the family Raphidascarididae)]. It does not differentiate putative hybrids of *A. simplex* (s. s.) and *A. pegreffii.*

The remaining larvae (280 individuals) were divided into four groups of 70 larvae each and cultured in 6-well plates (BD Biosciences, Warsaw, Poland) at a density of 10 larvae per well. The first group was directly subjected to in vitro culture with glucose (10 mg/mL of culture medium) for 24 h, while the second group served as a control culture without added sugar (abbreviated as L3 CTR and L3 GLU, which stands for the control and treated groups, respectively). The third and fourth groups were cultured in vitro to develop into L4 stage larvae as described by Iglesias et al. [13]. After 6 days, when the larvae had moulted to the L4 stage, they were subjected to in vitro culture with glucose (10 mg/mL of culture medium) for 24 h or control culture without added sugar (abbreviated as L4 CTR and L4 GLU, representing the control and treated groups, respectively). After all in vitro cultures, the larvae from the four groups (10 individuals/sample) were frozen at −80 °C (a total of 4 × 7 samples, giving a total of 28 samples) until the time of further analyses.

### Total RNA isolation and RNA-Seq

Total RNA was isolated from the sample sets (L3 CTR, L3 GLU, L4 CTR and L4 GLU; 28 samples in total) using the TRIzol reagent (Cat. No. 15596026, Invitrogen by Thermo Fisher Scientific, Waltham, Massachusetts, USA) in conjunction with the PureLink RNA Mini Kit (Cat. No. 12183018 A, Invitrogen by Thermo Fisher Scientific, Waltham, Massachusetts, USA) according to the manufacturer’s instructions. RNA quantity and integrity were checked using the Bioanalyzer 2100 and the RNA 6000 Nano LabChip Kit (Cat. No. 5067–1511, Agilent, CA, USA). Only high-quality RNA samples with a RIN number > 7.0 were used to generate the sequencing library (L3 CTR – 4 samples, L3 GLU – 3 samples, L4 CTR – 4 samples, and L4 GLU – 3 samples; in total 14 samples). Approximately 5 µg of total RNA was used to remove ribosomal RNA using the Ribo-Zero Gold rRNA Removal Kit (Cat. No. MRZG12324, Illumina, San Diego, USA) according to the manufacturer’s instructions. After removal of the ribosomal RNAs, the remaining RNAs were fragmented (Cat. No. E6150S, NEBNext® Magnesium RNA Fragmentation Module, New England Biolabs, Ipswich, MA, USA) into short fragments using divalent cations at high temperature (94 ℃, 5-7 min). Subsequently, the cleaved RNA fragments were reverse transcribed with SuperScriptTM II Reverse Transcriptase (Cat. No. 1896649, Invitrogen, Waltham, MA, USA) to generate cDNA, which was then used to synthesise U-labelled, second-stranded DNAs with *E. coli* DNA polymerase I (CatNo. m0209, New England Biolabs, Ipswich, MA, USA), RNase H (Cat. No. m0297, New England Biolabs, Ipswich, MA, USA) and dUTP solution (Cat. No. R0133, Thermo Fisher Scientific, Waltham, MA, USA). A-base was then added to the blunt ends of each strand to prepare them for ligation with the indicated adapters. Each adapter contained a T-base overhang for ligation of the adapter to the fragmented A-tailed DNA. The dual-index adapters were ligated to the fragments, and size selection (300-600 bp) was performed using AMPureXP beads (Cat. No. A63881, Beckman Coulter, Indianapolis, Indiana, USA). After treatment of the U-labelled secondary-stranded DNA with the heat-labile UDG enzyme (CatNo. m0280, NEB, New England Biolabs, Ipswich, MA, USA), the ligated products were amplified by PCR under the following conditions: initial denaturation at 95 °C for 3 min; 8 cycles of denaturation at 98 °C for 15 s, annealing at 60 °C for 15 s and extension at 72 °C for 30 s; and then final extension at 72 °C for 5 min. The average insert size of the final cDNA libraries was 300 ± 50 bp. Finally, we performed 2 × 150 bp paired-end sequencing (PE150) on an Illumina Novaseq™ 6000 (LC BioTechnology CO., Ltd., Hangzhou, China) according to the manufacturer's recommended protocol.

### Expression profiling

The quality of the sequencing was assessed using FastQC software [14]. After RNA-Seq, Illumina adapters and poly-A tails were removed using Trimmomatic v.0.39 [15]. Reads shorter than 120 nucleotides and with an average Phred quality score below 20 were excluded from the dataset. High-quality FASTQ reads were mapped to the *Anisakis simplex* PRJEB496 reference genome from WormBase ParaSite using STAR v.2.7.11.a. [16]. The resulting BAM files were then used to create annotations with StringTie v2.2.1 [17]. The Ballgown library [18] was then used to quantify expression and perform differential expression analysis between L3 GLU vs. L3 CTR, L4 GLU vs. L4 CTR and L4 GLU vs. L3 GLU. The genes with low expression were excluded from the study following read counting during R scripting procedure. The gene-level FPKMs for each sample were estimated by Ballgown tool. The RNAs with a log_2_FoldChange (log_2_FC) greater than 1 and an adjusted *p-*value (*p*-adj) less than 0.05 were considered statistically significant. Subsequently, the identified transcripts were categorized into three groups: differentially expressed genes (DEGs), differentially expressed long non-coding RNAs (DELs), and other RNAs. The gene descriptions were assigned using the biomaRt package [19] and ENSEMBL annotations [20]. Pearson correlations between the FPKM values for DEGs and DELs were determined using the cor function from the stats package in the R environment. The absolute threshold values for this coefficient (Pearson Correlation Coefficient) were > 0.7.

### Alternative splicing

The transcriptome fasta file was generated using genomic fasta format and the StringTie annotation (GTF file) with gffread v0.12.7 software [21]. Then, high-quality fastq files were remapped to transcriptome sequences using Salmon v.0.13.1 [22]. The program SUPPA v.2 [23] was then used to predict transcriptomic changes in alternative splicing events (ASEs). ASEs were categorized into seven main types: alternative 5' splice sites (A5), alternative 3' splice sites (A3), mutually exclusive exons (MX), retained introns (RI), skipped exons (SE), alternative first (AF) and alternative last (AL) exons. Events with differences in Percent Spliced In (ΔPSI) values between two experimental conditions ΔPSI > 0.1 and FDR < 0.05 were considered statistically significant. This PSI metric represents proportion of transcripts that include a specific splicing event. Differential alternative splicing events (DASEs) were identified by comparison: L3 GLU vs. L3 CTR, L4 GLU vs. L4 CTR, and L4 GLU vs. L3 GLU.

### Functional annotations

All DEGs were further analyzed for enrichment in Gene Ontology (GO) annotations utilizing the g:profiler v.0.2.2 R package [24]. Ontological annotations were assigned to significant genes for biological processes (BP), cellular components (CC), and molecular functions (MF) categories. Enrichment analysis was subsequently utilized to discover GO categories regulated by significant molecules, with an adjusted P-value threshold of < 0.05.

### Real-time PCR

The mRNA level of selected genes was determined by real-time PCR according to the general approach described by Polak et al. (2022) [12] and Stryiński et al. (2024) [25], with modifications. Total RNA was extracted using the Trizol reagent in combination with the PureLink RNA Mini Kit (Cat. No. 12183018 A, Invitrogen by Thermo Fisher Scientific, Waltham, MA, USA) according to the manufacturer’s guidelines. The cDNA synthesis was performed using the Applied Biosystems™ High-Capacity cDNA Reverse Transcription Kit (Thermo Fisher Scientific, Vilnius, Lithuania, Cat. No. 4374966) according to the protocol provided. Specific primers were designed using Primer3Plus software based on sequences from GenBank (Table S1). The qPCR reactions were prepared using Applied Biosystems™ PowerUp™ SYBR™ Green Master Mix (Thermo Fisher Scientific, Vilnius, Lithuania, Cat. No. A25780) containing 5 μL of 2X Master Mix, 500 nM of each primer, 10 ng of cDNA and nuclease-free water to a final volume of 10 μL. Amplifications were carried out on a QuantStudio™ 3 Real-Time PCR System (Applied Biosystems™, Thermo Fisher Scientific Inc., Waltham, MA, USA) in four technical replicates. The reaction conditions were as follows: initial denaturation at 95 °C for 10 min, followed by 40 cycles of 15 s at 95 °C, 60 s at at 60 °C, and 30 s at 72 °C. Reaction specificity was verified by melting curve analysis. The relative mRNA concentrations were quantified using the comparative Pfaffl method [26]. The efficiency of all primers was above 90%. Changes were plotted against the untreated control and normalised against endogenous reference genes: actin (KP200883) and glyceraldehyde-3-phosphate dehydrogenase (KM496565) (RQ = 1). Results were expressed as means ± standard deviations (SD) and plotted on a log₁₀ scale. Statistical significance was determined using a two-tailed Student’s t-test in Prism 8 (GraphPad Software Inc., San Diego, CA, USA), with *p*-values interpreted as follows: 0.0332 (*), 0.0021 (**), 0.0002 (***), and < 0.0001 (****).

### Protein extraction, TMT labeling and reversed-phase fractionation

Proteins from the organic phase obtained from “total RNA isolation” were extracted from the collected samples i.e., from the L3 larvae of *A. simplex* cultured with (L3 GLU, *n* = 3) and without (L3 CTR, *n* = 2) glucose*,* and from the L4 larvae of *A. simplex* cultured with (L4 GLU, *n* = 3) and without (L4 CTR, *n* = 2) glucose, according to the manufacturer’s instructions (TRIzol reagent, Cat No 15596026, Invitrogen from Thermo Fisher Scientific, Waltham, Massachusetts, USA). The total protein concentration in the supernatants was measured using the bicinchoninic acid method (Pierce BCA Protein Assay Kit, Thermo Fisher Scientific, Waltham, MA, USA).

From all samples (10 samples in total), a total of 100 μg of protein was transferred to new tubes and an overnight acetone precipitation was performed (1.8 mL of cold acetone was added to each sample and incubated overnight at - 20 °C). Then the samples were centrifuged (15,000 × g, 10 min, 4 °C) and tryptic digestion was performed with simultaneous application of high intensity focused ultrasound (HIFU) as previously described by Stryiński et al. [27, 28].

The peptide concentration in each sample was determined by colorimetric analysis using the Quantitative Colorimetric Peptide Assay (Thermo Fisher Scientific, Waltham, MA, USA) according to the manufacturer’s instructions. The isobaric TMT 10-Plex labelling reagents (0.8 mg, Thermo Fisher Scientific, Waltham, MA, USA) were added to 100 μg of protein digest as described by Stryiński et al. [28]. As part of the experiment, the samples were labeled with TMT10 plex as follows: L3 C: 126, 127 N; L4 C: 127 C, 128 N; L3 GLU: 128 C, 129 N, 129 C, and L4 GLU: 130 N, 130 C, 131. The samples within the TMT set were combined in equal amounts in a new tube according to the manufacturer’s instructions. To increase the number of peptide identifications, eliminate interference from co-isolated ions, and achieve comparable results to the MS^3^-based methods, the combined sample was fractionated using a Pierce High-pH Reversed-Phase Peptide Fractionation Kit (Thermo Fisher Scientific, Waltham, MA, USA) as previously described [28]. The peptide concentration in each fraction was determined by colorimetric analysis using the Quantitative Colorimetric Peptide Assay (Thermo Fisher Scientific, Waltham, MA, USA) according to the manufacturer’s instructions. The fractions were then evaporated to dryness by vacuum centrifugation (SpeedVac concentrator, Thermo Fisher Scientific, Waltham, MA, USA). The samples were stored at −80 °C until further analysis.

### LC–MS/MS analysis and data processing

Peptide fractions (eight fractions in total) were acidified with 0.1% formic acid and analyzed by nLC-MS/MS using a Proxeon EASY-nLC II liquid chromatography system (Thermo Fisher Scientific, Waltham, MA, USA) coupled to an LTQ-Orbitrap Elite mass spectrometer (Thermo Fisher Scientific, Waltham, MA, USA) as previously described [29, 30]. All acquired MS/MS spectra were analyzed with SEQUEST-HT (Proteome Discoverer 2.4 package, Thermo Fisher Scientific, Waltham, MA, USA) against a reference proteome of *Anisakis simplex* (proteome UniProt ID: UP000267096; 20,779 entries). The following restrictions were applied: full tryptic cleavage with up to 2 missing cleavage sites and tolerances of 10 ppm for parent ions and 0.06 Da for MS/MS fragment ions. TMT-labeling (+ 229.163 Da at N-termini and lysine residues) and carbamidomethylation of cysteine (+ 57.021 Da) were defined as fixed modifications. The allowed variable modifications were methionine oxidation (+ 15.994 Da), acetylation (+ 42.011 Da) of the N-terminus of the protein and deamidation (+ 0.984 Da) of asparagine and glutamine. In addition, the search parameters included four maximum dynamic modification sites.

### Statistical analysis of proteomics data and visualizations

The results were subjected to statistical analysis to determine the peptide false discovery rate (FDR) using a Decoy database and the Target Decoy PSM Validator algorithm [31]. The FDR was kept below 1% and only proteins that met selected criteria were submitted for further analysis: a) master proteins, b) proteins with any abundance value in each sample group, and c) proteins with different protein IDs. Relative quantification was performed using the quantification mode and normalization was performed against the total peptide amount (Proteome Discoverer 2.4 package, Thermo Fisher Scientific, Waltham, MA, USA). After relative quantification, several filters were applied to obtain the final list of DRPs: a) at least a 1.5-fold change in normalized ratios and b) ANOVA and Tukey HSD post-hoc test (*p*-value < 0.05).

The identified DRPs were visualized in a volcano plot. Analysis of protein–protein interactions was performed by submitting the DRPs dataset to Cytoscape (v. 3.8.0.; NIGMS, Bethesda, MD, USA), a software platform for visualizing complex networks, and analyzed by stringApp (v. 1.5.1.) [32] as described before [33]. The Markov Cluster Algorithm (MCL) was used for clustering the obtained networks (inflation parameter = 3).

### ELISA assay

The enzyme-linked immunosorbent assay (ELISA) was performed for selected proteins to validate the LC–MS/MS-based method. The concentrations of ATP-dependent 6-phosphofructokinase (ATP-PFK), heparan sulphate (HS) and methylmalonyl-CoA epimerase (MCEE) were analyzed using ELISA kits from Enlibio Biotech Co, Ltd (Wuhan, China, Cat. No. EIA09433Ge, EIA05815Ge, EIA09872Ge), according to the manufacturer’s protocols. All the ELISA kits included a monoclonal antibody targeting a conserved/general epitope of the selected protein. The samples were analyzed in triplicate. The results were calculated by comparison with a standard curve. As the data met the assumptions of normality (Shapiro–Wilk test) and homogeneity of variances (Levene’s test), ordinary two-way ANOVA and Dunnett’s multiple comparison tests were used to analyze differences between quantitative values. Statistical significance was defined as a *p*-value ≤ 0.05 (GraphPad Prism software version 10, San Diego, CA, USA).

## Results

### Glucose-mediated transcriptional regulation: stage-specific changes in L3 and L4 gene expression

**Fig. 1:**
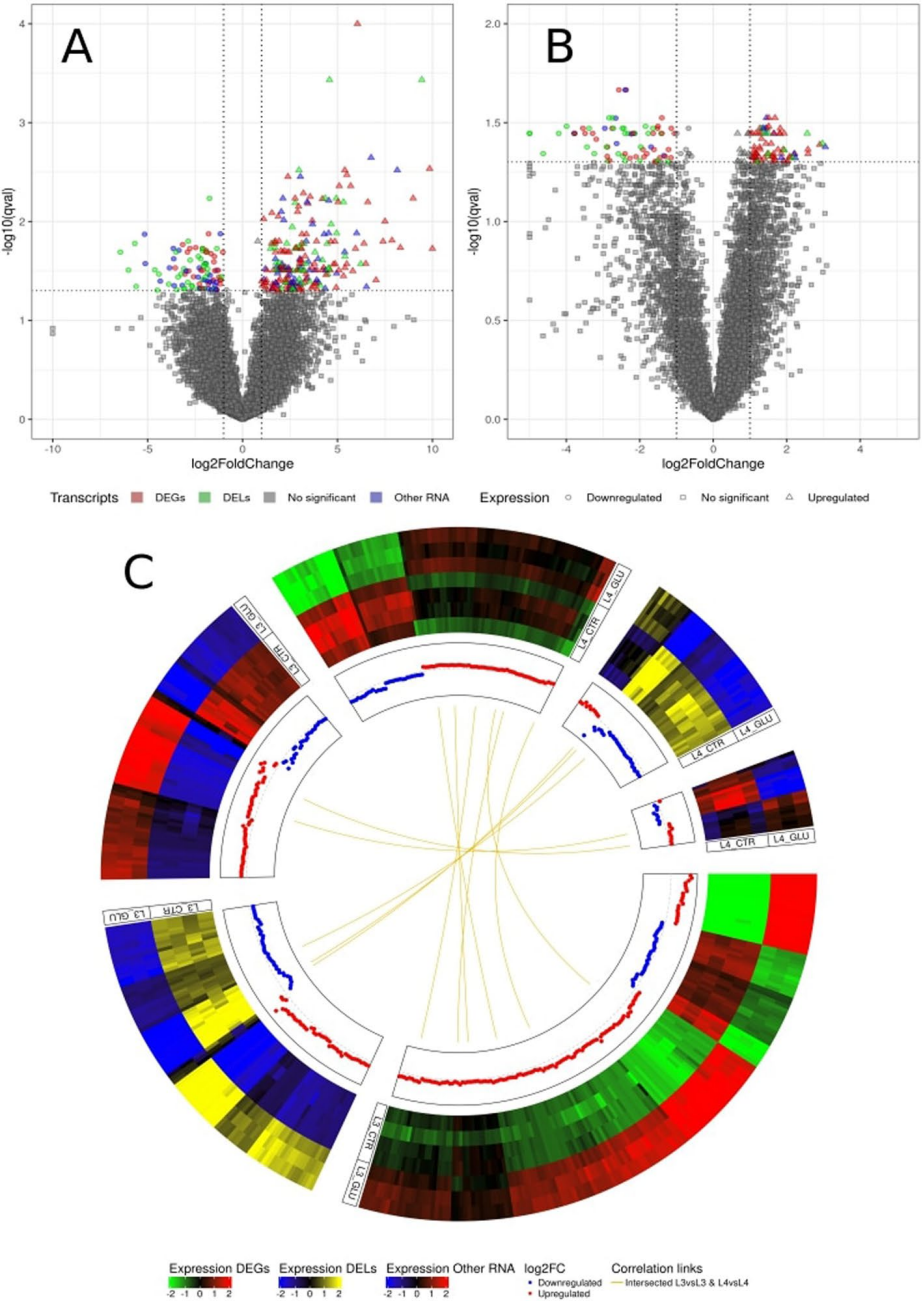
Analysis of glucose-induced gene expression changes in L3 and L4 stages. **A**, **B—**The volcano plot shows the relationship between -log_10_(padj) on the Y-axis and log_2_FoldChange on the X-axis for the L3 GLU *vs*. L3 CTR comparison (**A**) and the L4 GLU *vs*. L4 CTR comparison (**B**). DEGs are marked in red, DELs in green, other RNAs in blue, and statistically insignificant genes in gray. Additionally, downregulated genes are marked with a circle, statistically insignificant genes with a square, and upregulated genes with a triangle. **C—**A circular heatmaps presenting the associations between differentially expressed molecules in two comparisons. Heatmaps present the z-scored of FPKM expression values in six separate blocks (green–red scale for DEGs, blue-yellow scale for DELs and blue-red scale for Other RNA). The second track describes the log2FoldChange of upregulated (red) and downregulated (blue) molecules. The inner track presents the correlation: gold links between L3 GLU *vs*. L3 CTR and L4 GLU *vs*. L4 CTR comparison

In the L3 GLU *vs.* L3 CTR comparison, 317 RNAs were identified, with 96 being downregulated and 221 upregulated (Supplementary File 1, Table 1). In the L4 GLU *vs*. L4 CTR comparison, 144 RNAs were identified, with 71 being downregulated and 73 upregulated (Supplementary File 1, Table 2). Regarding the distribution of these molecules between different RNA groups, 157 DEGs, 84 DELs, and 76 other RNAs were identified in the L3 GLU *vs.* L3 CTR comparison (Fig. 1A), and 85 DEGs, 40 DELs, and 19 other RNAs were identified in the L4 GLU *vs.* L4 CTR comparison (Fig. 1B). Gene expression analysis between stages under the glucose treatment revealed that 620 statistically significant RNAs were identified in the L4 GLU *vs.* L3 GLU, with 285 molecules being downregulated and 335 upregulated (Supplementary File 1, Table 3). In terms of the distribution of statistically significant molecules into specific groups, 314 RNAs were assigned into DEGs, 163 into DELs, and 143 into other RNAs (Fig. 1C).

**Fig. 2:**
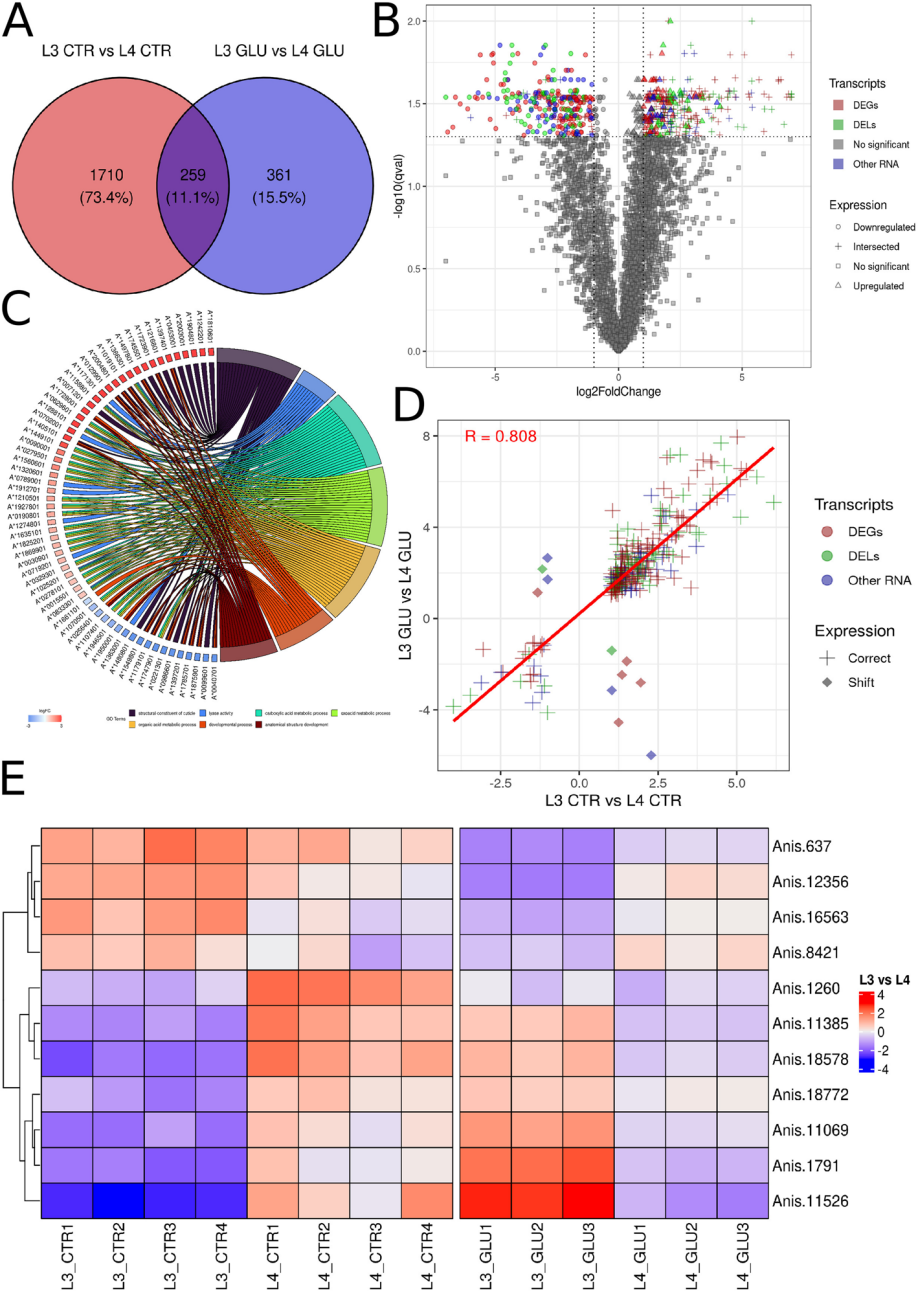
Comparative gene expression profiling of L3 and L4 stages and under glucose treatment. **A—**The red area in the Venn diagram represents significant DE genes in the L4 CTR *vs*. L3 CTR comparison, the blue area represents significant DE genes in the L4 GLU *vs*. L3 GLU comparison, and the middle-shaded area corresponds to genes common to both comparisons. **B—**The volcano plot shows the transcripts expressed in L4 GLU vs. L3 GLU, with -log_10_(p-adj) on the Y-axis and log_2_FoldChange on the X-axis. DEGs are marked in red, DELs in green, other RNAs in blue, and statistically insignificant genes in gray. Additionally, downregulated genes are marked with a circle, genes common to the L4 CTR *vs*. L3 CTR and L4 GLU *vs*. L3 GLU comparisons with a cross, statistically insignificant genes with a square, and upregulated genes with a triangle. **C—**The circos chart shows the assignment of L4 GLU vs. L3 GLU DEGs to GO processes, where each line represents a connection, and each color represents a different process. **D—**The scatter plot shows the relationship between log_2_FoldChange in the L4 GLU *vs*. L3 GLU comparison and log_2_FoldChange in the L4 CTR *vs*. L3 CTR comparison. Genes with the same expression pattern are marked with a cross, and genes with the opposite pattern with a rhombus. **E—**The heatmap shows the expression of 11 genes with reversed expression. The control comparison is shown on the left, and the glucose comparison on the right. Blue color symbolizes negative z-score values, and red color symbolizes positive z-score values

To investigate the direct effect of glucose on expression patterns, the differences in expression between L4 CTR and L3 CTR were identified. There were 1,969 genes with differential expression (Supplementary File 1, Table 4), of which 259 overlapped with the L4 GLU vs. L3 GLU comparison (Fig. 2A, B) and 11 showed a reversal expression, among which were 5 DEGs, 2 DELs, and 4 other RNAs (Fig. 2D, E; Supplementary File 1, Table 5).

The GO analysis showed that organonitrogen compound biosynthetic process (GO:1,901,566) in the L3 GLU *vs*. L3 CTR comparison, and translation (GO:0006412), peptide biosynthetic process (GO:0043043), and amide metabolic process (GO:0043603) in the L4 CTR *vs*. L4 GLU comparison were the most enriched biological processes (Supplementary Figs. 1, 2; Supplementary File 1, Table 6). The differentially expressed molecules in L4 GLU *vs*. L3 GLU were found to be involved in the following biological processes: carboxylic acid metabolic process (GO:0019752), oxoacid metabolic process (GO:0043436), anatomical structure development (GO:0048856), and developmental process (GO:0032502) (Fig. 2C; Supplementary File 1, Table 6).

**Fig. 3:**
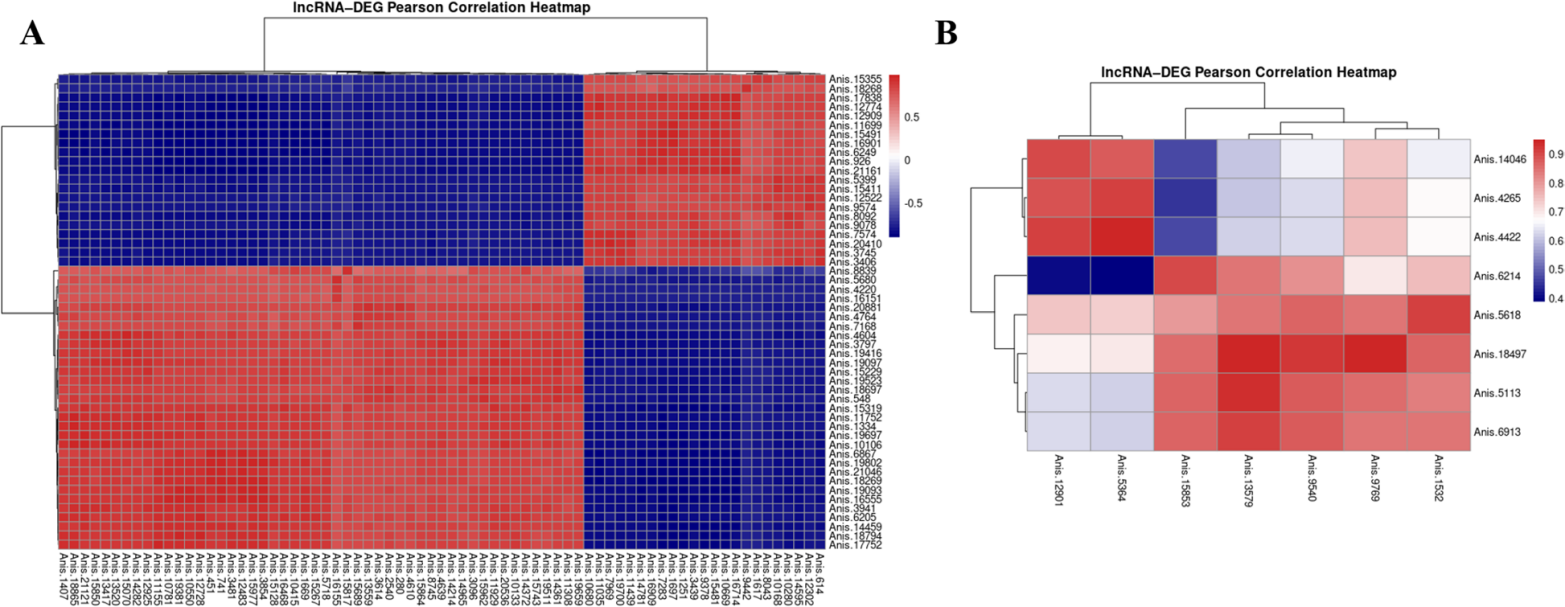
The results of Pearson’s correlations analysis between DEGs and DELs. Panel **A** shows correlation matrix for transcripts identified in comparison of L3 GLU vs L3 CTR, and panel **B** for transcripts identified in comparison of L4 GLU vs L4 CTR

The results of the correlation between differentially expressed genes and long non-coding RNAs revealed 73 DEGs strongly correlated (Pearson correlation > 0.9) with 52 DELs in the L3 GLU (Fig. 3A). In turn, the analysis of L4 GLU revealed a strong correlation between 7 DEGs and 8 DELs (Fig. 3B). The comparison of L4 GLU vs L3 GLU showed 109 DEGs correlated with 63 DELs (Supplementary Fig. 3). Such results may have implications for the potential regulation of protein-coding genes by non-coding factors (Supplementary File 1, Tables 7—9).

The RNA-Seq approach results were confirmed using real-time PCR, which was used to demonstrate the expression of 17 randomly selected RNAs. Molecules for the validation were selected according to their functionality, expression levels, and distribution across samples. There was a strong agreement (Pearson's r = 0.663, *p*-value = 0.00077) between the RNA expression data obtained by real-time PCR and RNA-Seq (Supplementary Figs. 4 and 5).

### Analysis of differential alternative splicing events in L3 and L4 stages under glucose treatment

**Fig. 4:**
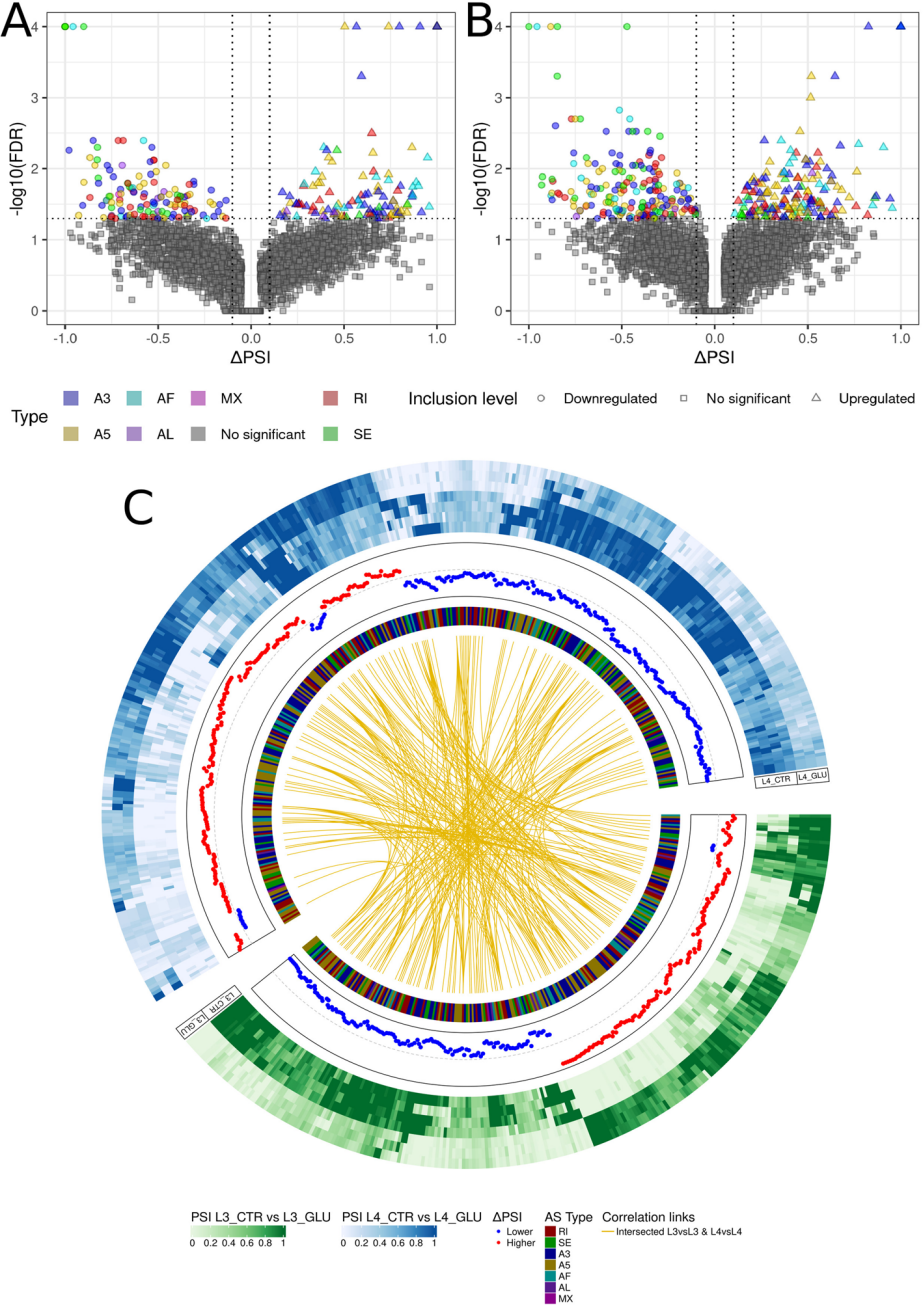
Comprehensive analysis of differential alternative splicing events in L3 and L4 larvae. **A**, **B**—A volcano plot illustrates the distribution of splicing events in the L3 GLU *vs*. L3 CTR comparison (**A**) and the L4 GLU *vs*. L4 CTR comparison (**B**). Each point corresponds to a different DASEs. Each splicing event is marked with a different color. The shape of the point on the volcano plot depends on the ΔPSI regulation: downregulated events are represented by circles, upregulated events by triangles, and non-significant events by squares. **C**—A circos plot presents a comparison of PSI in L3 GLU *vs*. L3 CTR (green heatmap) and L4 GLU *vs*. L4 CTR (blue heatmap), where a darker shade of color indicates higher PSI. A scatter plot indicates the level of regulation, with downregulated events (blue) and upregulated events (red). The inner heatmap shows the type of event: red for RI, green for SE, blue for A3, yellow for A5, cyan for AF, purple for AL, and magenta for MX. The center connecting lines symbolize splicing events in the same gene

**Fig. 5:**
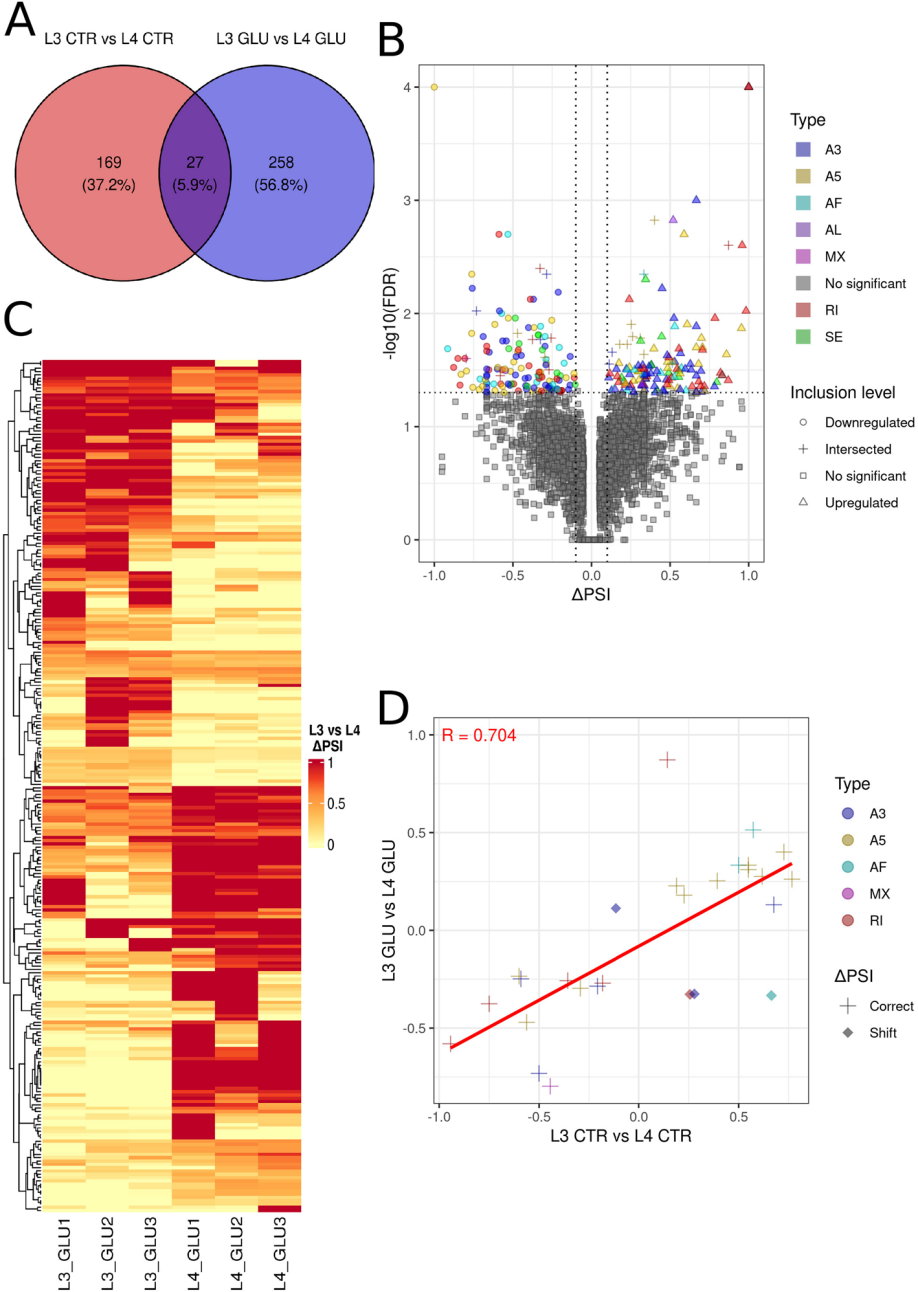
Differential alternative splicing events across L3 and L4 stages induced by glucose treatment.** A**—A Venn diagram illustrates the distribution of DASEs in the L4 CTR *vs*. L3 CTR comparison (red) and the L4 GLU *vs*. L3 GLU comparison (blue), with the central shaded area representing the common DASEs between the two comparisons. **B**—A volcano plot displays the distribution of all splicing events in the L4 GLU *vs*. L3 GLU comparison, where different splicing types are indicated by colors. Additionally, different point shapes denote the following: circles for downregulated events, squares for statistically non-significant events, triangles for upregulated events, and crosses for events shared with the L4 CTR *vs*. L3 CTR comparison. **C**—A heatmap shows the PSI values across various samples from the L4 GLU *vs*. L3 GLU comparison, with yellow indicating low inclusion, orange indicating medium inclusion, and red indicating the highest inclusion. **D**—A scatter plot represents the correlation between PSI values of splicing events, with the y-axis representing the L4 GLU *vs*. L3 GLU comparison and the x-axis representing the L4 CTR *vs*. L3 CTR comparison. Different types of splicing events are marked by different colors, while consistency in ΔPSI is indicated by a cross, and a shift is indicated by a diamond

To investigate the influence of glucose on alternative splicing events (ASE) in L3 and L4 stages, two analyses of ASEs were conducted, one identifying differential alternative splicing events (DASEs) between stages under the glucose influence, and second between control larvae (Figs. 4 and 5; Supplementary File 1, Table 10).

Analysis within each developmental stage, compared to the glucose-treated groups, revealed 258 and 375 DASEs in the comparisons L3 GLU *vs*. L3 CTR and L4 GLU *vs*. L4 CTR, respectively.

In the L3 GLU *vs*. L3 CTR comparison 133 DASEs had a ΔPSI lower than −0.1 and 125 had a ΔPSI greater than 0.1 (Fig. 4A, C). In the L4 GLU *vs*. L4 CTR comparison, 196 DASEs had a ΔPSI lower than −0.1 and 179 had a ΔPSI greater than 0.1 (Fig. 4B, C). The distribution of DASEs by type was as follows: for L3 GLU *vs*. L3 CTR, 81 A3, 70 A5, 26 AF, 9 AL, 1 MX, 46 RI, and 25 SE were identified; for L4 GLU *vs*. L4 CTR, 117 A3, 102 A5, 38 AF, 5 AL, 1 MX, 62 RI, and 50 SE were identified (Fig. 4; Supplementary File 1, Table 10). Of all the DASEs identified, 241 were found within annotated RNAs in the L3 GLU *vs*. L3 CTR comparison, and 365 were found within annotated RNAs in the L4 GLU *vs*. L4 CTR comparison (Fig. 5C).

A comparison, L4 CTR *vs*. L3 CTR, was performed to examine the actual impact of glucose on the *A. simplex* transcriptome. It was hypothesized that DASEs common to both the L4 GLU *vs*. L3 GLU and L4 CTR *vs*. L3 CTR comparisons resulted from transcriptomic differences between the L3 and L4 stages, rather than from the direct effect of glucose. The analysis of the L4 CTR *vs*. L3 CTR comparison revealed 196 DASEs, where 27 were also found in the L4 GLU *vs*. L3 GLU comparison (Fig. 5A, B). Within the L4 GLU *vs*. L3 GLU comparison, 285 DASEs were identified, among which 140 were exhibited with a ΔPSI lower than −0.1, and 145 were shown with a ΔPSI greater than 0.1 (Fig. 5B, C). The distribution of DASEs by type was as follows: 92 A3, 73 A5, 31 AF, 5 AL, 1 MX, 59 RI, and 24 SE were identified (Fig. 5B; Supplementary File 1, Table 10).

Four DASEs were observed to exhibit reversed ΔPSI values between the L4 GLU *vs*. L3 GLU and L4 CTR *vs*. L3 CTR comparisons. These included Anis.16916 (AF), Anis.16945 (NADAR domain-containing protein; A3), Anis.8960 (spectrin alpha chain; RI), and Anis.9954 (dynamin GTPase; A3). In each case, the directionality of splicing changes was opposite between the control and glucose-treated conditions across the developmental stages examined (Fig. 5D; Supplementary File 1, Table 11).

### Differentially regulated proteins in A. simplex larvae under the glucose treatment

**Fig. 6:**
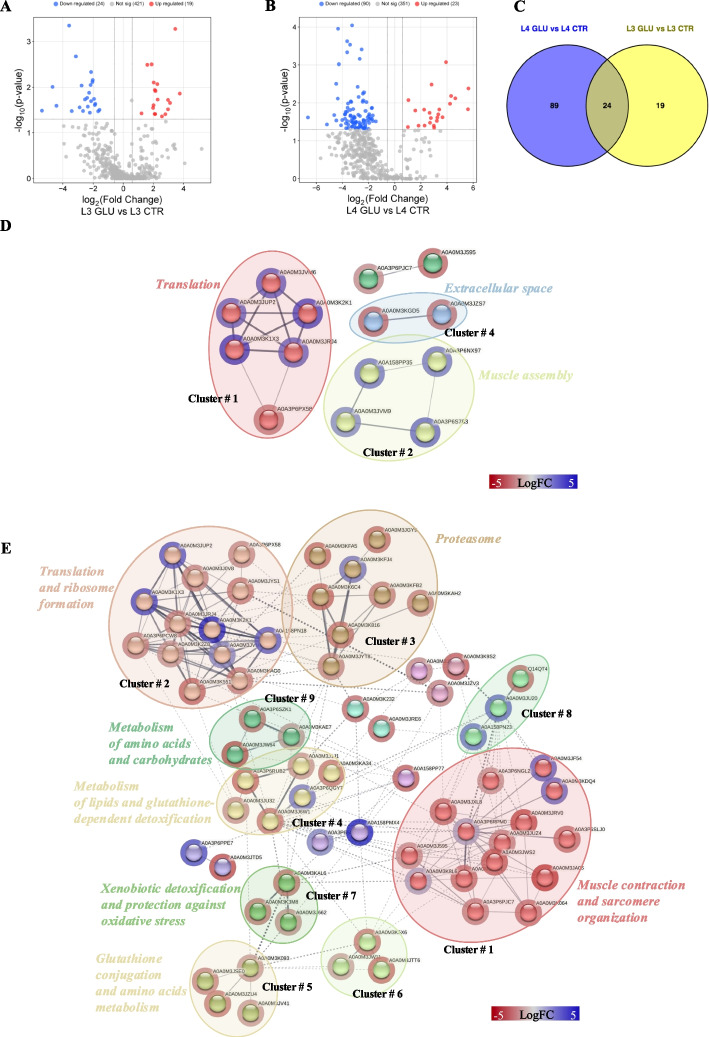
Changes in the proteomes of L3 and L4 stages induced by glucose treatment. **A**, **B –** The Volcano plots to visualize the DRPs of L3 and L4 *A. simplex* larvae treated with glucose (FC ≥ 1.5; *p*-value < 0.05). **C**—Analysis of the distribution of common and unique DRPs between different larval stages. **D**, **E**—Protein–protein interactions network analysis of DRPs identified in this study. Interactions of DRPs identified in L3 larvae after glucose treatment (**D**), and in L4 larvae after glucose treatment (**E**). Red circle borders – up-, green circle borders – downregulated proteins. The Markov Cluster Algorithm (MCL) was used for the clustering the network (inflation parameter = 3). Different colors inside the circles are different clusters. Selected clusters are described by Name and numbers in the figure. The results of PPI analyses are present in Supplementary File 2, Tables 11–15

The proteome of *A. simplex* larvae treated with glucose was analyzed by LC–MS/MS. Statistical analysis of the data led to the generation of Volcano plots to visualize the DRPs (Fig. 6; Supplementary File 2, Table 1). The proteome of L3 larvae was slightly altered when cultured with glucose (Fig. 6A). Twenty-four proteins were down-regulated, and 19 proteins were up-regulated compared to larvae cultured without glucose (FC ≥ 1.5; *p*-value < 0.05; Supplementary File 2, Tables 2, 3). Proteomic analysis of L4 larvae cultured with glucose (Fig. 6B) showed that 113 proteins were differentially regulated compared to the control, with 90 proteins down-regulated and 23 proteins up-regulated (FC ≥ 1.5; *p*-value < 0.05; Supplementary File 2, Tables 4, 5). The comparison of L3 and L4 larvae cultured without treatment showed 15 DRPs (FC ≥ 1.5; *p*-value < 0.05; Supplementary File 2, Tables 6, 7). On the other hand, comparison of the proteomes of L3 and L4 larvae cultured with glucose revealed 35 DRPs, of which 5 were more abundant in the L4 stage and 30 in the L3 stage (FC ≥ 1.5; *p*-value < 0.05; Supplementary File 2, Tables 8, 9). The specific proteins that were differentially regulated can be found in Supplementary File 2, Tables 2—9.

Analysis of the distribution of common and unique DRPs between different larval stages showed that the same 24 proteins, associated with translation and ribosome formation, were affected in both L3 and L4 larvae treated with glucose (Fig. 6C, Supplementary File 2, Table 10).

The protein–protein interactions analysis was also performed (Supplementary File 2, Table 11). Fourteen proteins, out of 43 DRPs in the proteome of L3 larvae when cultured with glucose, constituted a network with 18 interactions (PPI enrichment *p*-value = 0.0161, Fig. 6D). The MCL clustering revealed 4 groups of proteins, one of which contained as many as 6 interacting proteins (cluster no. 1, Fig. 6D). This cluster grouped proteins involved in the process of protein translation (Supplementary File 2, Table 12). The most interactive proteins were *40S ribosomal protein S6* (5 interactions, cluster no. 1; UniProt ID: A0A0M3K1X3) and *60S ribosomal protein L4* (5 interactions, cluster no. 1; UniProt ID: A0A0M3JRJ4) (Supplementary File 2, Table 13).

A total of 68 DRPs, out of 113 DRPs identified the proteome of L4 larvae cultured with glucose, constituted a very complex and strongly interactive network (217 interactions) (Fig. 6E, Supplementary File 2, Table 11). The MCL clustering revealed 16 clusters (PPI enrichment *p*-value = 2.22e-16, Supplementary File 2, Table 14). These clusters included proteins involved in processes such as muscle contraction and sarcomere organization (cluster no. 1), translation, ribosome binding, and regulation of protein synthesis (cluster no. 2), proteasome and ubiquitin-dependent protein degradation (cluster no. 3), detoxification with glutathione (cluster no. 4), and glutathione conjugation (cluster no. 5), as well as xenobiotic detoxification and protection against oxidative stress (cluster no. 7) (Fig. 6E, Supplementary File 2, Table 14). The top five most interactive proteins were *fatty acid-binding protein homolog 9* (18 interactions, cluster no. 4; UniProt ID: A0A0M3J6W1), *EF-hand domain-containing protein* (18 interactions, cluster no. 1; UniProt ID: A0A3P6RPM0), *60S ribosomal protein L4* (15 interactions, cluster no. 2; UniProt ID: A0A0M3JRJ4), *60S ribosomal protein L44* (14 interactions, cluster no. 2; UniProt ID: A0A0M3JVM6), and proteasome subunit alpha type (13 interactions, cluster no. 2; UniProt ID: A0A0M3JYT8) (Fig. 6E, Supplementary File 2, Table 15).

The LC–MS/MS approach results were validated using ELISA assay, which was used to demonstrate the abundance of 3 randomly selected proteins. There was an agreement between the protein abundance data obtained by ELISA assay and LC–MS/MS (Supplementary Fig. 6).

### Relationship between changes at the proteins and RNAs level

**Fig. 7:**
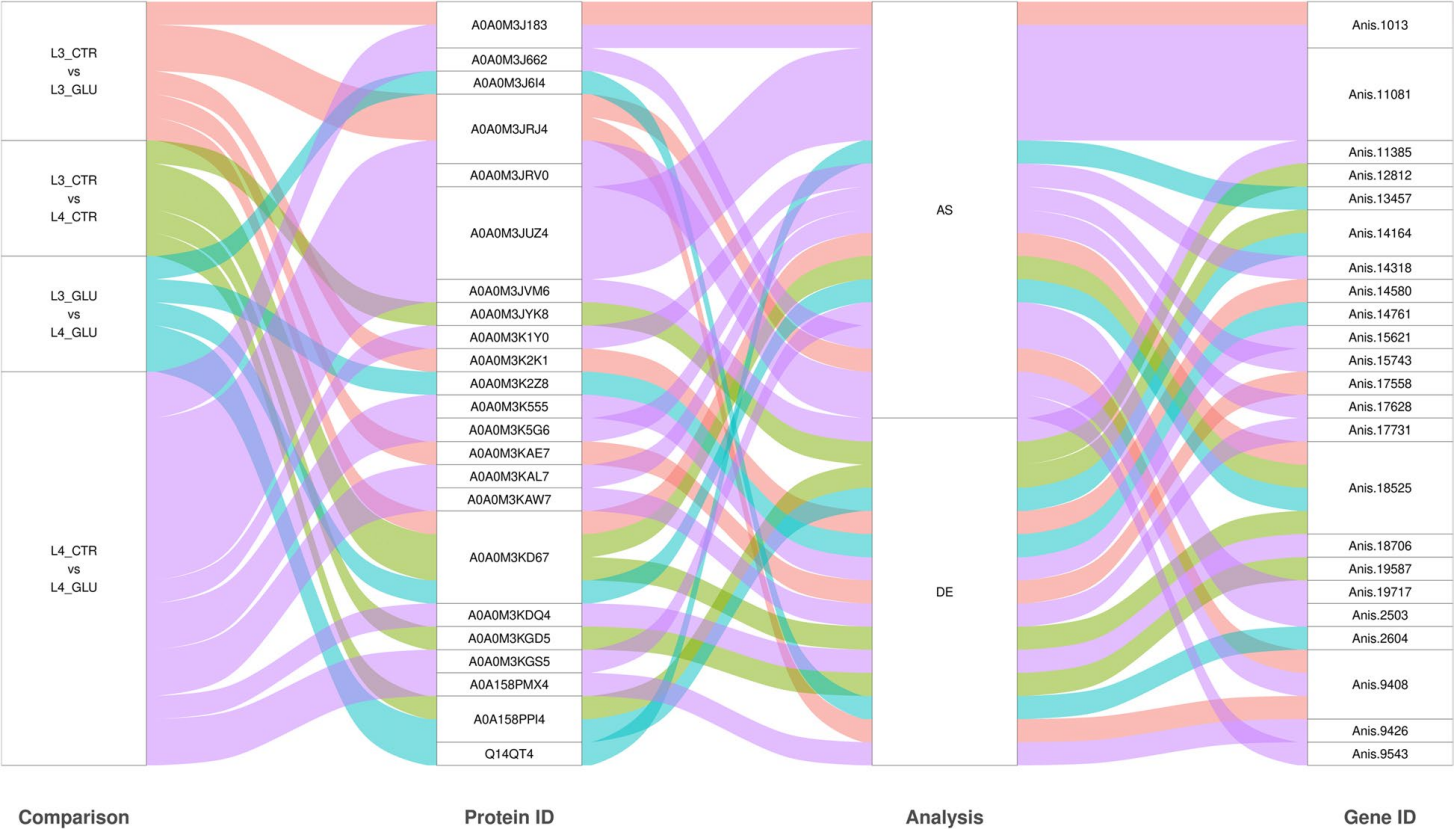
The Sankey diagram shows the relationships between identified proteins and DEGs in DE analysis and DAS in AS methodology. The "Comparison" column presents comparisons between conditions: red—L3 GLU *vs*. L3 CTR, green – L4 CTR *vs*. L3 CTR, blue – L4 GLU *vs*. L3 GLU and purple—L4 GLU *vs*. L4 CTR. The "Protein ID" column contains unique protein identifiers. The "Analysis" column specifies whether DEGs and DAS were detected in DEG or AS analysis. The "Gene ID" column provides identifiers of the most similar genes according to blast assigned by StringTie software

In all four comparisons, thirty-three correlations between transcriptomics and proteomics data were identified. Among these, 15 targets showed consistent changes at both the gene expression and protein activity levels (Fig. 7, Supplementary File 1, Table 12). Additionally, 18 molecules exhibited fluctuations in splicing and protein activity, where 12 primarily driven by glucose treatment of the L4 stage larvae (Fig. 7, Supplementary File 1, Table 13). Two molecules, myosin tail 1 domain-containing protein (A0A0M3KD67, ASIM_0001891901) and 60S ribosomal protein L4 (A0A0M3JRJ4, ASIM_0001035801) exhibited changes in both transcriptomic levels (DEGs and DASEs) and after the translation process (DRPs). Specifically, 6 associations (3 related to DASEs, 3 to DEGs) were found in the L3 GLU *vs*. L3 CTR comparison, 5 associations (3 related to DASEs and 2 to DEGs) in the L4 GLU *vs*. L3 GLU comparison, and 17 associations (5 related to DASEs, 12 to DEGs) in the L4 GLU *vs*. L4 CTR comparison (Fig. 7, Supplementary File 1, Tables 12, 13).

## Discussion

Intestinal parasitic infections (IPIs) are among the most critical public health problems worldwide. According to the World Health Organization (WHO), 1.5 billion people, i.e., 24% of the world population, are affected by IPIs, primarily by soil-transmitted helminths such as *Ascaris lumbricoides*, *Trichuris trichiura*, *Ancylostoma duodenale*, and *Necator americanus* [34]. *Anisakis simplex*, a parasitic nematode of the Anisakidae family, also contributes to this burden, particularly in regions with high consumption of raw or undercooked fish, such as Japan, and increasingly in Western countries including Spain, Italy, and France [35, 36]. As the only fish-borne parasites known to trigger allergic reactions in humans, anisakids pose a unique biological hazard in seafood worldwide [3, 4].

Like many gastrointestinal helminths, *A. simplex* larvae thrive in anaerobic environments where carbohydrates serve as primary energy sources [37–39]. Among these, glucose, glycogen, and trehalose play dominant roles in supporting larval survival and development. Although previous studies have characterized glucose metabolism in other nematodes [5, 6], the direct influence of exogenous glucose on the L3 and L4 developmental stages of *A. simplex* s.s. had not been elucidated until now.

Modern high-throughput techniques enable comprehensive investigation and analysis at multiple levels of metabolic regulation—including the transcriptome, post-transcriptional modifications, and proteome. A transcriptomic study of a closely related species, *A. pegreffii*, showed that mitochondrial enzymes were most highly expressed in L3 larvae, while polyubiquitin and collagen-related genes predominated in L4 [40]. Similarly, earlier work on the Spanish population of *A. simplex* s.s. revealed proteomic differences between L3 and L4 larvae associated with energy metabolism, oxidative stress, cellular transport, and signaling pathways [27]. However, the specific effect of glucose as an external factor in the host environment has not yet been investigated.

Reasonable intake of carbohydrates is essential for the development of gastrointestinal nematode larvae. Previous studies have shown that sugars exert a stronger physiological effect on nematodes than lipids at low to moderate concentrations, while lipids can be damaging at high concentrations (100–400 mmol/L) [41]. The glucose concentration of 10 mg/mL (approximately 55 mmol/L) used in this study was chosen based on earlier findings, where this level resulted in maximal differentiation in glucose transporter activity (e.g., FGT/GLUT). Moreover, 50 mmol/L treatments of sucrose, fructose, or glucose have been shown to significantly prolong lifespan and delay mortality in *C. elegans* [42].

The results of this study highlight that the developmental stage has a greater impact on transcriptomic and proteomic profiles than glucose treatment alone. This conclusion is supported by statistical analyses, which showed that the extent of differences in the expression of various RNA types—including DEGs, DASEs, and DELs—as well as protein levels (DRPs), was substantially greater between developmental stages than between control and glucose-treated groups within the same stage. This is particularly evident in the differential expression of genes and proteins associated with structural remodeling, energy metabolism, and translation machinery. Specifically, L4 larvae showed more extensive transcriptional and proteomic remodeling, consistent with their more developed and functional digestive system [7]. The differences observed likely result from both the structural functionality of the intestine and its ability to absorb nutrients from the host environment. In contrast, L3 larvae have a collapsed, non-functional gut, limiting glucose uptake.

Glucose has a subtle, stage-dependent effect. Not all DEGs between stages remain significant under glucose treatment. The reduction in DEGs in L4 GLU vs L3 GLU may indicate that glucose modulates, but does not completely override, developmental transcriptional programs. This observation aligns with the finding that only a small number of genes displayed divergent expression patterns under glucose between stages, suggesting that developmental stage exerts a stronger influence than glucose exposure.

Transcriptomic analyses revealed characteristic activities of metabolic pathways in both L3 and L4 stages. In related parasitic nematodes such as *A. suum* and *N. americanus*, differentially expressed clusters of metabolomic modules have been observed depending on the larval stage, particularly with increasing energy demands in L4 and adult forms. These include pathways such as glycolysis, glutathione metabolism, and CoA biosynthesis [43]. Similarly, in *A. simplex*, differences in intestinal structure and functionality between L3 and L4 likely affect the absorption of nutrients from the host's intestinal environment. In our study, genes involved in carbohydrate metabolism (e.g., glycogen binding subunit 76 A, trehalase, ATP-dependent 6-phosphofructokinase) were differentially expressed between stages (L4 CTR vs. L3 CTR). Comparisons also revealed that more genes linked to lipid metabolism were differentially regulated across larval stages than in response to glucose treatment. This included the activation of genes involved in the degradation of odd-chain fatty acids and branched-chain amino acids, such as methylmalonyl-CoA mutase and methionine synthase. These enzymes play central roles in the propionate catabolism and one-carbon cycle, respectively. The recently described propionate shunt pathway in *C. elegans* [44] aligns with our findings and supports the presence of similar regulatory processes in *A. simplex*.

Only 11 genes under glucose exposure displayed divergent expression patterns between larval stages, with four annotated: a SAM domain-containing protein (SAMSN1), 60S ribosomal protein L44, a Col_cuticle_N domain-containing protein, and a globin-like protein (GLB-1). SAMSN1 belongs to a novel gene family encoding scaffold proteins with SH3 and SAM (sterile alpha motif) domains [45], which may also bind to RNA [46]. Alongside ribosomal proteins, they could be involved in mRNA transport, stability, and translation. The glucose-induced expression of a globin-like protein (GLB-1) under hypoxic conditions in *C. elegans* [47] may similarly reflect roles in oxygen-sensitive metabolism in *A. simplex*.

Genes encoding allergenic proteins such as actin, tropomyosin, and heat shock proteins (HSP90) were also glucose-responsive. Additionally, chaperone protein HSP90 was co-expressed with calponin, a calcium-binding protein, suggesting that glucose may trigger an endoplasmic reticulum (ER) stress response. This pattern mirrors the glucose-regulated protein GRP94 observed in bovine filarial parasite, *Setaria cervi*, where it maintains ER integrity and protein quality control [48–51].

The results of the correlation analysis between DEGs and DELs showed that previously described DEGs such as Col_cuticle_N domain-containing protein and GLB-1 (L4 GLU vs. L3 GLU), as well as HSP90 (L4 GLU vs. L4 CTR), may be regulated by non-coding RNAs. Moreover, in all three comparisons (Supplementary File 1, Tables 7–8), DELs were shown to correlate with DEGs involved in saccharide transport, e.g., sugar transporter SWEET (Anis.14595), transmembrane 9 superfamily (Anis.12901), and MFS domain-containing protein (Anis.10634). These results may indicate a role of DELs in the regulation of DEGs associated with glucose metabolism.

One of the study's most notable contributions is the integration of alternative splicing events (ASE) with gene and protein expression data. Alternative splicing is a powerful mechanism that increases protein diversity and functional adaptation in multicellular organisms [52, 53]. In nematodes, the stage-specific splicing patterns could support adaptation to host immune defenses and environmental stressors [54, 55]. These spliced isoforms can have distinct molecular functions and biological roles and may be differentially expressed among tissues, life cycle stages or environmental conditions [52]. Revisiting existing datasets with a cDNA-specific, splice-aware, assembly protocol would provide a far more accurate impression of ASE in parasitic nematodes, a factor that can have important practical implications with respect to pathogenesis, drug susceptibility/resistance, and vaccine development [55].

Differential alternative splicing events (DASEs) were identified in this study in dynamin GTPase (A3), spectrin alpha (RI), hemicentin (A3, A5, SE, MX), tropomyosin (SE), and myosin (A5) (Supplementary File 1, Tables 10, 11 and 13). Dynamin GTPase affects endocytosis and cytoskeletal remodeling and is essential for development and locomotion [56]. Spectrin dynamically translates changes in actin structure into epithelial morphogenesis during embryogenesis in *C. elegans* (SMA-1) [57]. Hemicentin, encoded by *him-4*, contributes to ECM mechanics and anchoring of hemidesmosomes in the epidermis [58–60]. Tropomyosin and myosin isoforms regulate the force and speed of muscle contraction, contributing to larval mobility and potentially modulating allergenicity at the molecular level [48].

Proteins belonging to the basement membrane proteoglycans were also differentially regulated in *A. simplex* under glucose conditions (e.g., collagen triple helix repeat protein, laminin-like protein epi-1). These proteins play essential roles in tissue morphogenesis, cuticle patterning, and tissue homeostasis [61–63]. Basement membranes, once viewed as static scaffolds, are now recognized as dynamic regulators of cell behavior and tissue organization. Emerging evidence in *C. elegans* suggests that ECM remodeling and basement membrane turnover may also influence longevity, adding another layer of physiological regulation.

The results of the proteomic analysis showed that glucose significantly affected proteins related to ribosome assembly, muscle contraction, and metabolic enzymes. For instance, 40S ribosomal protein S6 and 60S ribosomal protein L4 showed were highly interactive proteins under glucose treatment, supporting enhanced translational activity. L4 larvae in particular showed proteomic responses associated with glutathione-mediated detoxification, sarcomere organization, and protein degradation. Analysis integrating transcriptomic and proteomic levels identified 33 overlapping signals, with 15 showing consistent changes. These differences may result from post-transcriptional regulation, variable mRNA stability, or differences in translation efficiency and protein degradation rates. Examples include myosin tail 1domain-containing protein and 60S ribosomal protein L4, both demonstrating concurrent differential expression (DEGs), alternative splicing (DASEs), and changes at the protein level (DRPs). This alignment across regulatory levels reflects precise molecular coordination, particularly under glucose treatment.

These findings suggest that glucose in *A. simplex* regulates transcription factors and enzymes governing cytoskeletal dynamics, ECM structure, translation, and energy metabolism. Overall, the multilevel analysis suggests that larval stage—more than glucose availability—determines metabolic and structural remodeling in *A. simplex*. This is supported by the fact that in L3 larvae, the inactive intestine and lack of active glucose transporters may prevent systemic metabolic changes in response to glucose.

## Conclusions

This study presents the first comprehensive multi-omics analysis examining the effects of glucose on *A. simplex* s.s. across two developmental stages, L3 and L4. The data reveal that developmental stage exerts a more substantial influence than glucose exposure on gene and protein expression profiles. However, glucose still modulates several pathways related to translation, cytoskeletal remodeling, extracellular matrix (ECM) reorganization, and energy metabolism.

By integrating RNA expression (DEGs and DELs), alternative splicing events, and proteomic data, the study highlights a complex regulatory network that underlies larval development and environmental adaptation. This multi-layered regulation involves isoform-specific expression, structural protein modulation, and energy-related enzyme shifts—particularly in response to glucose availability. Importantly, understanding how high glucose levels induce systemic molecular changes will enhance the current knowledge of the molecular mechanisms of nutrition in this parasite. The findings also underscore the importance of ECM plasticity, cytoskeletal dynamics, and translational control in the development and pathogenicity of *A. simplex* s.s. Furthermore, this study demonstrates the value of combined transcriptomic and proteomic approaches in elucidating intricate biological processes.

## Supplementary Information

Supplementary Material 1. Supplementary Figure 1. Circular plot presents the associations of DEGs from L3 GLU vs. L3 CTR comparison to Gene Ontology (GO) processes. Each color corresponds to an all significantly enriched GO terms, and each line represents a single association. The scale bar near the gene names indicates the log2FoldChange value of the gene, with blue representing negative values (downregulated) and red representing positive values (upregulated).

Supplementary Material 2. Supplementary Figure 2 Circular plot presents the associations of DEGs from L4 GLU vs. L4 CTR comparison to Gene Ontology (GO) processes. Each color corresponds to a all significantly enriched GO terms, and each line represents a single association. The scale bar near the gene names indicates the log2FoldChange value of the gene, with blue representing negative values (downregulated) and red representing positive values (upregulated).

Supplementary Material 3. Supplementary Figure 3. The results of correlations analysis between DEGs and DELs identified in comparison of L4 GLU vs L3 GLU.

Supplementary Material 4. Supplementary Figure 4. The barplots demonstrate the comparison of log2FoldChange between the RT-PCR method and RNA-seq. The red bars represent the log2FoldChange after RNA-seq expression profiling analysis, while the blue bars represent the log2FoldChange after the RT-PCR based methodology. The y-axis shows the log2FoldChange, while the x-axis shows the gene names. Each comparison has its own segment, from top to bottom: L3 GLU vs L3 CTR, L4 GLU vs L3 GLU, and L4 GLU vs L4CTR.

Supplementary Material 5. Supplementary Figure 5. The results of correlations analysis between RT-PCR method and RNA-seq. Selected genes were marked with different color for each comparison. The y-axis shows the log2FoldChange obtained by the RNA-seq method, while the x-axis shows the log2FoldChange of validated DEGs measured by the qPCR method. Details can be found in Supplementary File 1, 2 and 3.

Supplementary Material 6. Supplementary Figure 6. The results of ELISA assay.

Supplementary Material 7. Supplementary File 1 - results of RNA Seq analysis

Supplementary Material 8. Supplementary File 2 - results of LC-MS/MS analysis

Supplementary Material 9. Table S1. The list of primers used for Real-time PCR.

## Data Availability

The transcriptomic data were submitted to the European Nucleotide Archive (ENA) and are available under the dataset number PRJEB84918. The proteomic data are publicly and freely available in the MassIVE repository (www.massive.ucsd. edu) under the dataset number MSV000097135. Other data results are available upon reasonable request to the corresponding author.
