# Supplementary material for: Coupled transcriptome and proteome analysis of L3 and L4 developmental stages of Anisakis simplex s. s.: insights into target genes under glucose influence
Source: BMC Genomics. 2025 Sep 29;26:866. doi: 10.1186/s12864-025-12068-w (PMC12482602; doi:10.1186/s12864-025-12068-w)
Supplement: Supplementary file 1 — Supplementary Material 1. Supplementary Figure 1. Circular plot presents the associations of DEGs from L3 GLU vs. L3 CTR comparison to Gene Ontology (GO) processes. Each color corresponds to an all significantly enriched GO terms, and each line represents a single association. The scale bar near the gene names indicates the log2FoldChange value of the gene, with blue representing negative values (downregulated) and red representing positive values (upregulated). [file 12864_2025_12068_MOESM1_ESM.pdf]

Supplementary Figure 1. Circular plot presents the associations of DEGs from L3 GLU vs. L3 CTR comparison to Gene Ontology (GO) processes.

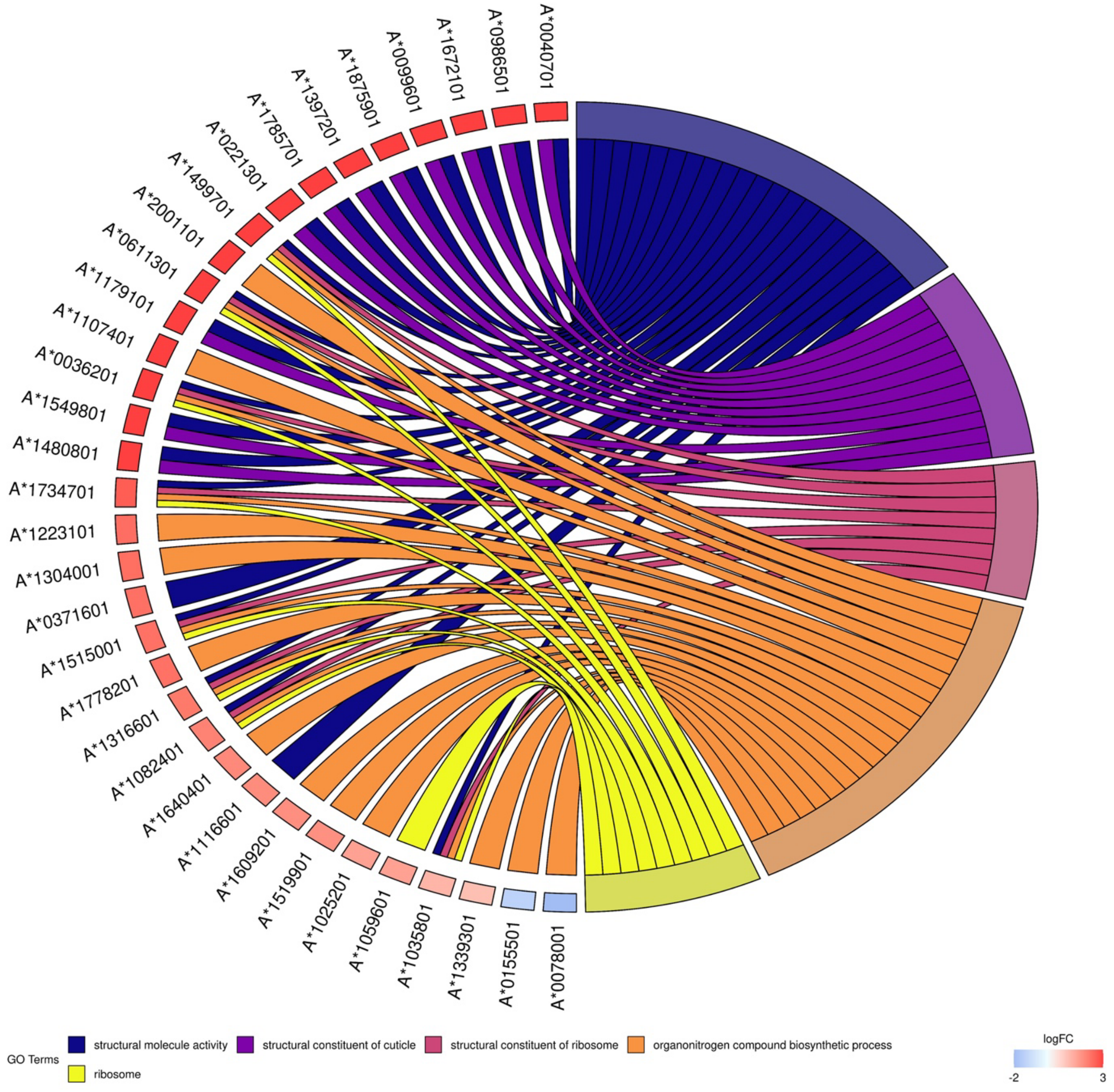

Each color corresponds to an all significantly enriched GO terms, and each line represents a single association. The scale bar near the gene names indicates the log2FoldChange value of the gene, with blue representing negative values (downregulated) and red representing positive values (upregulated).
