# Supplementary material for: Coupled transcriptome and proteome analysis of L3 and L4 developmental stages of Anisakis simplex s. s.: insights into target genes under glucose influence
Source: BMC Genomics. 2025 Sep 29;26:866. doi: 10.1186/s12864-025-12068-w (PMC12482602; doi:10.1186/s12864-025-12068-w)

Supplementary Figure 3. The results of correlations analysis between DEGs and DELs identified in comparison of L4 GLU vs L3 GLU.

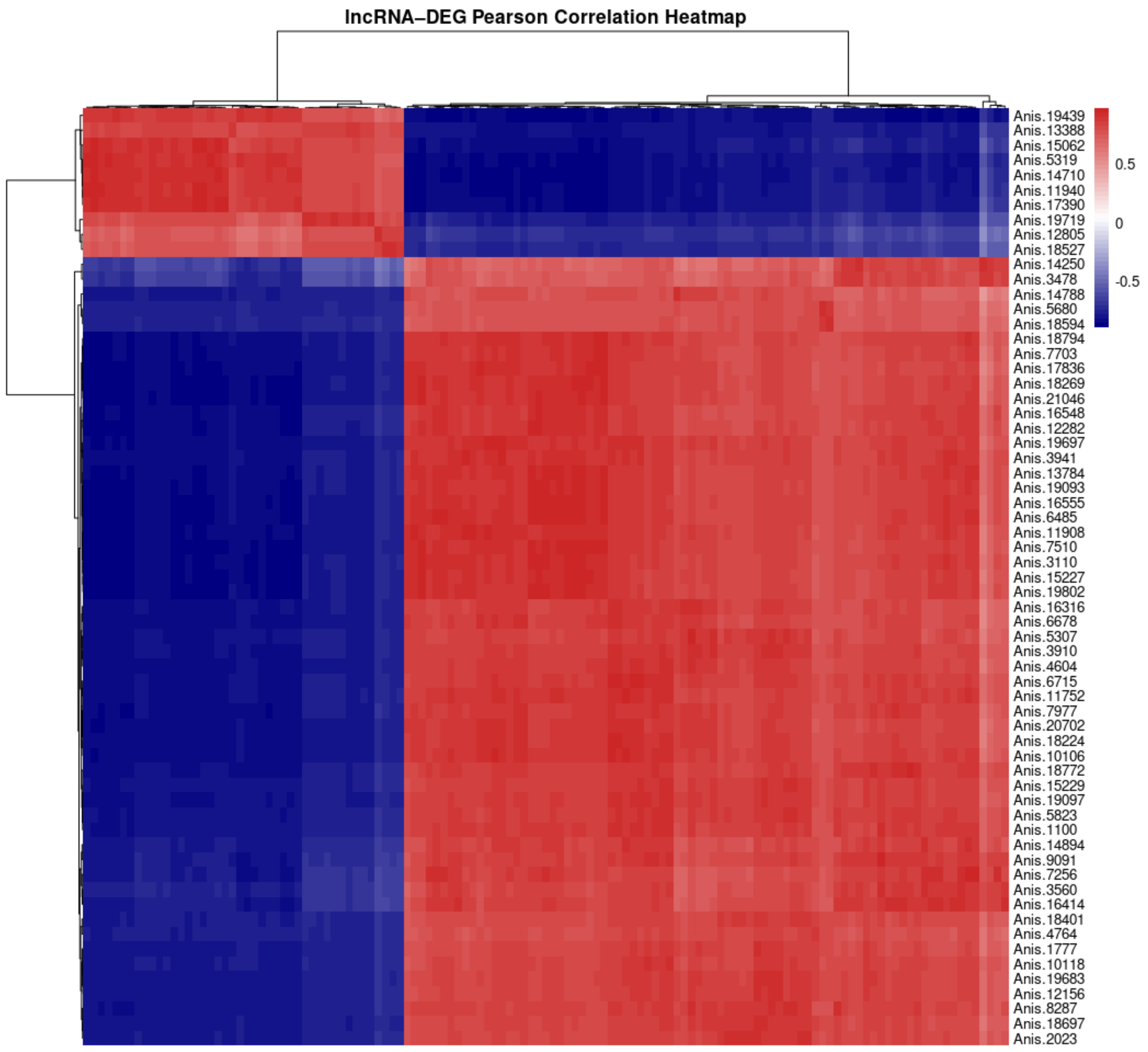

Supplement: Supplementary file 3 — Supplementary Material 3. Supplementary Figure 3. The results of correlations analysis between DEGs and DELs identified in comparison of L4 GLU vs L3 GLU. [file 12864_2025_12068_MOESM3_ESM.pdf]
