# Supplementary material for: Coupled transcriptome and proteome analysis of L3 and L4 developmental stages of Anisakis simplex s. s.: insights into target genes under glucose influence
Source: BMC Genomics. 2025 Sep 29;26:866. doi: 10.1186/s12864-025-12068-w (PMC12482602; doi:10.1186/s12864-025-12068-w)
Supplement: Supplementary file 4 — Supplementary Material 4. Supplementary Figure 4. The barplots demonstrate the comparison of log2FoldChange between the RT-PCR method and RNA-seq. The red bars represent the log2FoldChange after RNA-seq expression profiling analysis, while the blue bars represent the log2FoldChange after the RT-PCR based methodology. The y-axis shows the log2FoldChange, while the x-axis shows the gene names. Each comparison has its own segment, from top to bottom: L3 GLU vs L3 CTR, L4 GLU vs L3 GLU, and L4 GLU vs L4CTR. [file 12864_2025_12068_MOESM4_ESM.pdf]

Supplementary Figure 4. The barplots demonstrate the comparison of log2FoldChange between the RT-PCR method and RNA-seq.

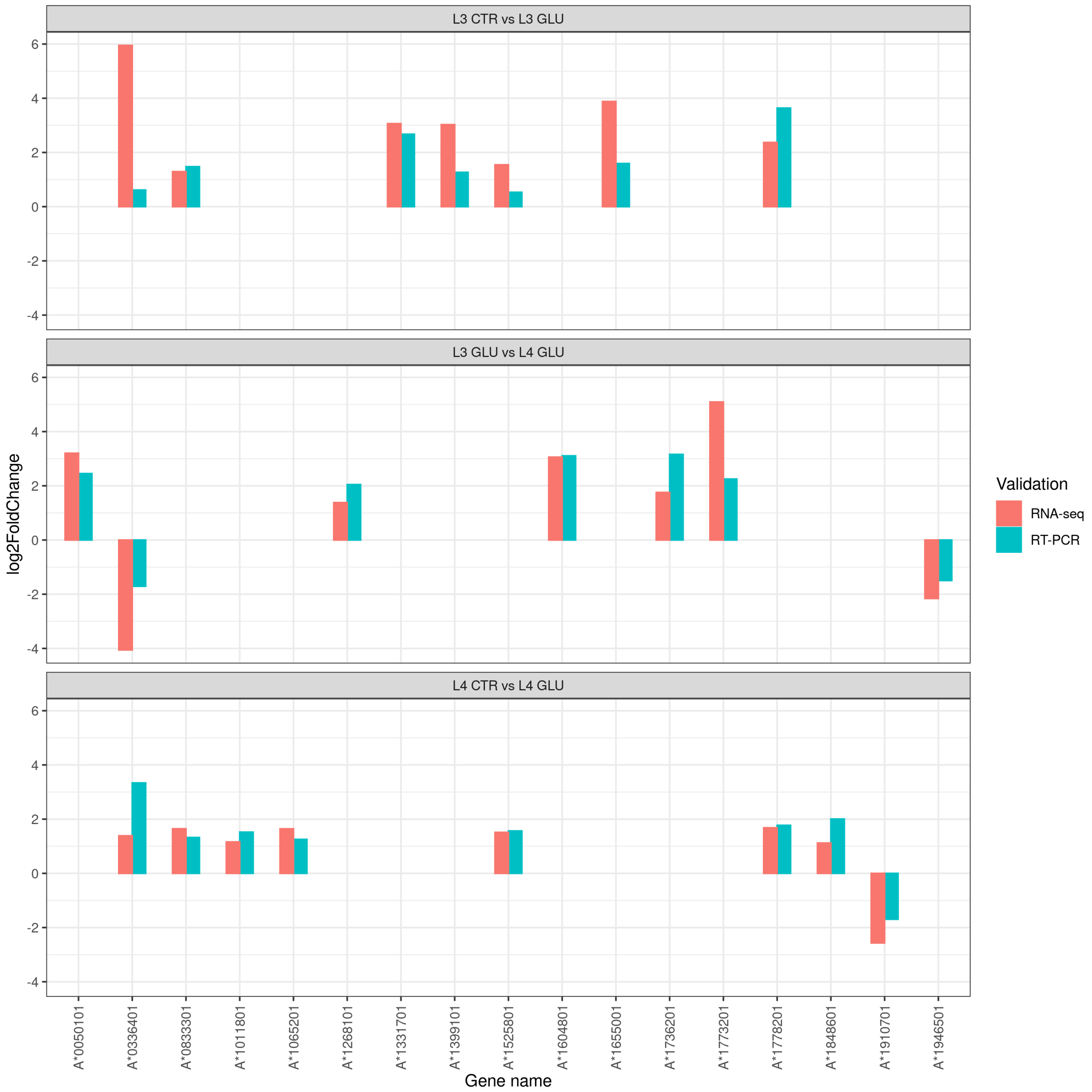

The red bars represent the log2FoldChange after RNA-seq expression profiling analysis, while the blue bars represent the log2FoldChange after the RT-PCR based methodology. The y-axis shows the log2FoldChange, while the x-axis shows the gene names. Each comparison has its own segment, from top to bottom: L3 GLU vs L3 CTR, L4 GLU vs L3 GLU, and L4 GLU vs L4CTR.
