# Supplementary material for: Coupled transcriptome and proteome analysis of L3 and L4 developmental stages of Anisakis simplex s. s.: insights into target genes under glucose influence
Source: BMC Genomics. 2025 Sep 29;26:866. doi: 10.1186/s12864-025-12068-w (PMC12482602; doi:10.1186/s12864-025-12068-w)
Supplement: Supplementary file 5 — Supplementary Material 5. Supplementary Figure 5. The results of correlations analysis between RT-PCR method and RNA-seq. Selected genes were marked with different color for each comparison. The y-axis shows the log2FoldChange obtained by the RNA-seq method, while the x-axis shows the log2FoldChange of validated DEGs measured by the qPCR method. Details can be found in Supplementary File 1, 2 and 3. [file 12864_2025_12068_MOESM5_ESM.pdf]

Supplementary Figure 5. The results of correlations analysis between RT-PCR method and RNA-seq.

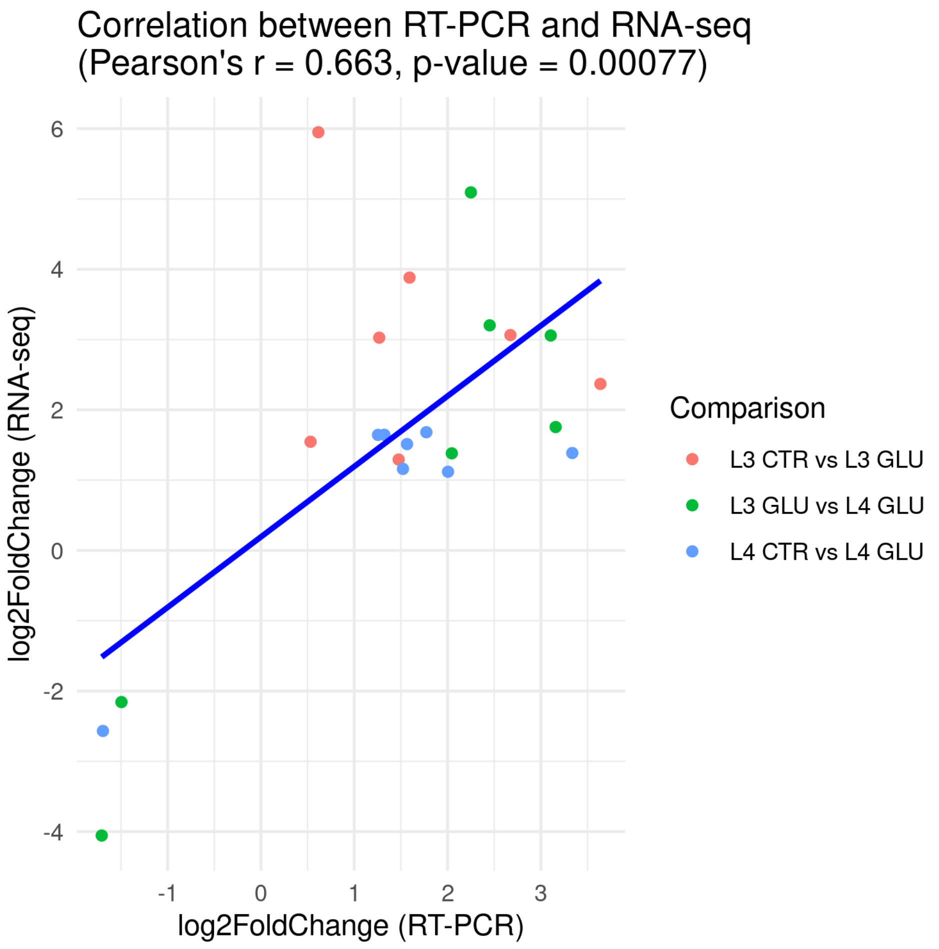

Selected genes were marked with different color for each comparison. **The y-axis shows the log2FoldChange obtained by the RNA-seq method, while the x-axis shows the log2FoldChange of validated DEGs measured by the qPCR method.** Details can be found in Supplementary File 1, 2 and 3.
