# Supplementary material for: Coupled transcriptome and proteome analysis of L3 and L4 developmental stages of Anisakis simplex s. s.: insights into target genes under glucose influence
Source: BMC Genomics. 2025 Sep 29;26:866. doi: 10.1186/s12864-025-12068-w (PMC12482602; doi:10.1186/s12864-025-12068-w)
Supplement: Supplementary file 6 — Supplementary Material 6. Supplementary Figure 6. The results of ELISA assay. [file 12864_2025_12068_MOESM6_ESM.pdf]

Supplementary Figure 6. The results of ELISA assay.

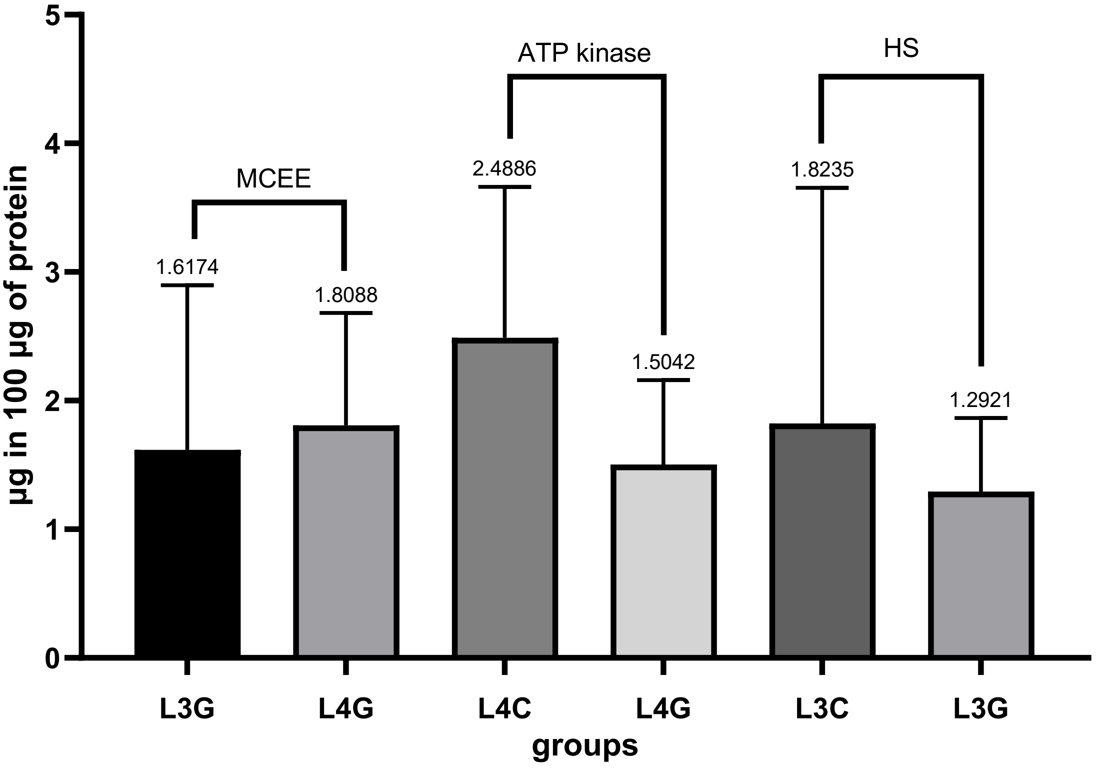

MCEE-methylmalonyl-CoA epimerase, **ATP-PFK**- ATP-dependent 6-phosphofructokinase, **HS**-heparan sulphate. Statistical significance was defined as a *p*-value ≤ 0.05.
