# Supplementary material for: Coupled transcriptome and proteome analysis of L3 and L4 developmental stages of Anisakis simplex s. s.: insights into target genes under glucose influence
Source: BMC Genomics. 2025 Sep 29;26:866. doi: 10.1186/s12864-025-12068-w (PMC12482602; doi:10.1186/s12864-025-12068-w)
Supplement: Supplementary file 9 — Supplementary Material 9. Table S1. The list of primers used for Real-time PCR. [file 12864_2025_12068_MOESM9_ESM.docx]

Table S1. The list of primers used for Real-time PCR.

| Name | No. accession GenBank | Forward and Reverse sequence: (5' to 3') | Product size (bp) |
| --- | --- | --- | --- |
| Hydroxyacyl-coenzyme A dehydrogenase | ASIM_0001946501 | F-ACGACACTAAGGCCCAAGAC  R-GTGTTCCAGTTCGTGAAGCG | 177 |
| Glycogen-binding subunit 76A | ASIM_0001848601 | F-GCCTCTTACGTGACCCCATT  R-CAAACGCCTTGCTTGAGTGT | 185 |
| Methylmalonyl-CoA mutase | ASIM_0001736201 | AAGACCGTGGACAATCCGAC GCCATACCAACGTCACCGTA | 174 |
| Trehalase | ASIM_0001655001 | GCATCCCGGCACAGAGTATT  ACACCGTTCCGTAAGAGCAG | 164 |
| Gelsolin-like protein 1 | ASIM_0001604801 | TGATGAGAATCTGTGGCGTCC  GCTCGTGTCCATCCAACACA | 176 |
| Myelin regulatory factor | ASIM_0001399101 | TTGAGATGCATGTGGTCGCT  CCCGCAAAATGCCAACTTGA | 188 |
| 10 kDa heat shock protein | ASIM_0001331701 | GCACCTGAGGTCAAGACCAA  TGTCACTTTTGTGCCTCCGT | 190 |
| Sulfate_transp domain | ASIM_0001268101 | GCCGTCGACCGAGTAATGAT AAGACCGCATATTGCAGCCA | 177 |
| Hsp90 chaperone protein kinase-targeting subunit | ASIM_0001065201 | CCTCCTTGAGCATCCGCATA  CGTTCGTAGCCAATGCATGA | 176 |
| Alanine--tRNA ligase | ASIM_0001011801 | TGCAATCCGTGATAAGGCGA  GCCTTACCACTGGCTCCTTT | 174 |
| Apolipoprotein B-100 | ASIM_0000050101 | CGCTTACGGAAATGACTGCG  CACTGCGTATCGCTCTCCAT | 179 |
| Troponin T | ASIM_0001910701 | GCATCTAGTGGCACTCCCAA  GCGCTTTCAGATCGTTCACC | 187 |
| Dolichyl-diphosphooligosaccharide--protein glycosyltransferase subunit 2 | ASIM_0001778201 | AGAGGCCAAGTCAGGGAATG  AACAGCATACTTGCCCGACA | 181 |
| Epimerase domain-containing protein | ASIM_0001773201 | ACGATACGGATTGCACTGGT  TCGGCGATTTTCTGCCTGAT | 187 |
| elongation factor 1-gamma | ASIM_0001532701 | GAGAGGTGGAATTCGCCACA  TCTTGGCTTGTTGCTGAGGT | 165 |
| protein CNPPD1 | ASIM_0001525801 | TTTGTTATGGCTCCAGGCGT  CTTTCCCGAGCCAGTGATGT | 181 |
| Calsequestrin | ASIM_0001143401 | AGCTGGCTGATGAGAACAGT  AGCAAACAACATGACGGCTG | 192 |
| fumarate hydratase | ASIM_0000833301 | GCACGGAGCACTGAATACGA  GCAGCAACCATAGTGAGAGC | 183 |
| Pepsin I3 domain | ASIM_0000336401 | TTGTCACCGAGCGAGATCAA  GTGCCAACGCTGTGGTAGAA | 196 |
| DB domain-containing protein | Anis.13630.1 | GGGAATCGTTGCTTGCGTTT  TAGTTTGACGACAGAGCGCA | 158 |
| Carboxypeptidase | Anis.621.1 | AGCGCTTGGTACCTGTGATT  CCGAGCATGAGTGTCCATGT | 193 |
| Actin gene | KP200883 | TGGAGTGGTGCTTGACTCAG  TCACGAACAATCTCACGCTC | 186 |
| Glyceraldehyde 4 phosphate dehydrogenase | KM496565 | CCGAAATCAAGTGGGGAGCA  TGGCAGCGTCGTACTTATCC | 183 |
